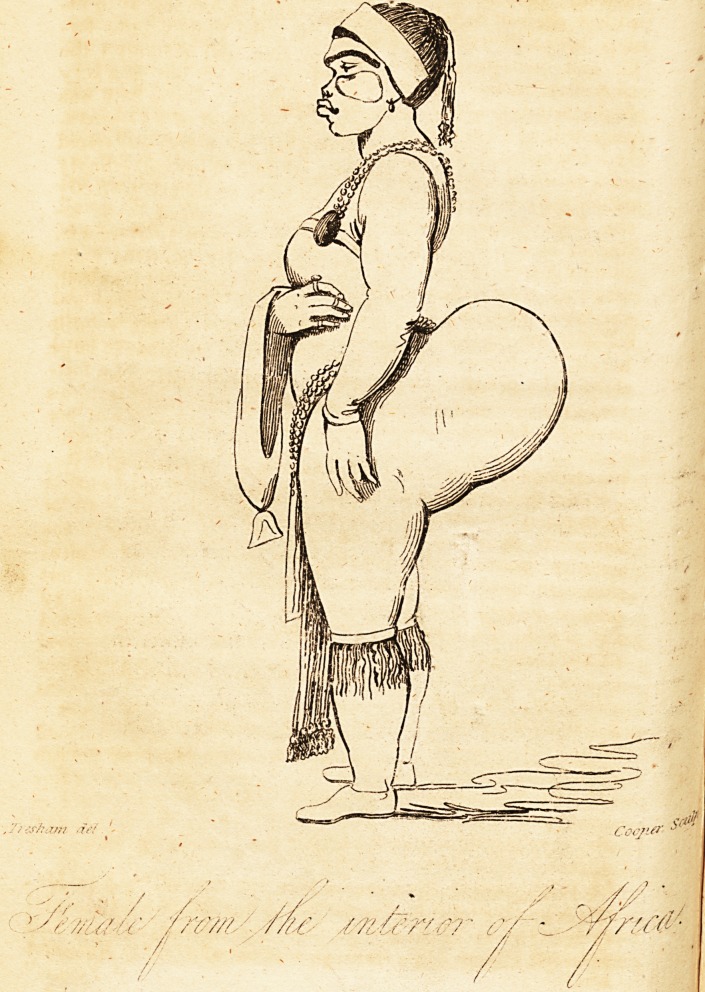# Singular Effect of Belladonna in an Uncommon Nervous Affection

**Published:** 1810-11

**Authors:** J. Bacot

**Affiliations:** Hospital, 1st Guards


*To the Editors of the Medical and Physical Journal.*

### *Singular Effect of Belladonna in an uncommon Nervous Affection.*

GENTLEMEN,

IN the month of January, 1809, Miss B. a healthy young lady, aged 29 years, received a severe blow upon the outer part of the fore arm, by the fall of a box lid. The pain was not particularly violent at the time, and a slight degree of ecchymosis was the only immediate consequence, which was soon dissipated by the application of some common remedies. In about a week, however, a fresh attack of pain was felt in the part that had been struck, and which increased very rapidly, extending upwards towards the axilla, and downwards in the direction of the supinator radii longus, but more particularly affecting the thumb and fore-finger; there was no appearance of inflammation or swelling upon the part, but a slight degree of tenderness upon pressure. Leeches, blisters, and a variety of topical applications were had recource to by the advice of a medical friend, but without affording any relief; on the contrary, the pain increased to a very violent degree, and the muscles of the fore-arm, particularly those employed in its rotation, became affected with occasional spasms, so that the arm was kept in motion for several successive hours. In this state she was recommended to come to London, and the complaint was so much aggravated by the journey, (only 36 miles), that the side of the neck and the head sympathised in the pain. Her general health did not appear to be much affected, her appetite continued good, the pulse natural, and all the evacuations regular.

During the months of March and April, the whole tribe of nervous and antispasmodic medicines were tried without effect. Opium produced only an alleviation of the pain, but had no influence upon the spasmodic contraction of the muscles; warm Baths, which were employed three times a week, had no better success, and Electricity certainly aggravated the disease: the only medicine that could be said to have been at all useful was the Cicuta; which, in large doses, certainly very much quieted the spasms, though it never overcame them entirely; it was however persisted in, till the head and stomach began to suffer from its use, and when discontinued on that account, the complaint resumed all its wonted violence, and, indeed, was perceptibly on the increase, the muscles of the arm itself taking on the same disposition, and the whole limb was thrown upwards with a most rapid and violent motion, for such a continuance of time as to completely exhaust the patient, whose only resource was in a large dose of Opium, which procured a very temporary remission of her sufferings. In this melancholy, and almost hopeless situation, on the 8th May, a solution of the Extr. Belladonnæ, in the proportion of ʒj of the extr. to ℥j of water, was applied in the direction of the nerves of the arm, at the suggestion of Mr. Copeland. When an ounce of this solution had been used, our patient began to complain of an uneasy sensation about the pit of the stomach;

Tresham del. Cooper Sculp

*Female from the interior of Africa.*

mach; but as this was not attributed to the application, it was continued during the following day, until a second ounce was expended: the oppression and anxiety about the præcordia, which, during this time, had been constantly increasing, then arose to a most distressing degree, attended with a sense of weight and pressure so intolerable, as to induce her to loosen all her garments; the pulse became feeble and intermitting, and the skin cold and clammy: the convulsive motion of the arm still continued, but in a very slight degree. Under these circumstance, a warm bath was prepared, and the patient was put in at a heat of 94 degrees. In a few minutes she fainted; and when taken out, was seized with a violent hysteric paroxysm, which left her exceedingly exhausted; from that moment her arm remained perfectly free from spasms, though a new disease seemed to be induced, no less formidable than the one so recently overcome. She appeared to have lost her memory entirely, the voice was very indistinct, and her articulation imperfect; indeed, she laboured under a complete amentia. Blisters were applied to the legs, and she was directed to take the volatile alkali, combined with the assafœtida and camphor in large doses; she persisted in this plan, and in the space of a fortnight these complaints began slowly to relax, her faculties gradually returned, and she recovered the perfect use of her speech; the original disease never shewed the least disposition to return; her state of debility was, however, extreme, the slightest agitation of spirits bringing on the most violent hysteric paroxysms; and it was not till the latter end of June that she was enabled to return to her residence in the country, where she has acquired her former state of health, and with it, the perfect use of the arm. It may not be improper to add, that this case was seen in its progress by Dr. Baillie, the late Mr. Ford, and several other gentlemen of the faculty.

(Signed) J. BACOT.

*Hospital, 1st Guards,*
*Oct. 20, 1810.*

## Figures and Tables

**Figure f1:**